# Behavioural Measures of Infant Activity but Not Attention Associate with Later Preschool ADHD Traits

**DOI:** 10.3390/brainsci11050524

**Published:** 2021-04-21

**Authors:** Amy Goodwin, Alexandra Hendry, Luke Mason, Tessel Bazelmans, Jannath Begum Ali, Greg Pasco, Tony Charman, Emily J. H. Jones, Mark H. Johnson, The BASIS/STAARS Team

**Affiliations:** 1 Department of Forensic and Neurodevelopmental Sciences, Institute of Psychiatry, Psychology & Neuroscience, King’s College London, London SE5 8AF, UK; 2 Department of Experimental Psychology, University of Oxford, Oxford OX2 6GG, UK; 3 Centre for Brain and Cognitive Development, Department of Psychological Sciences, Birkbeck, University of London, London WC1E 7HX, UK; ublmas001@mail.bbk.ac.uk (L.M.); ubjbeg001@mail.bbk.ac.uk (J.B.A.); ubejon01@mail.bbk.ac.uk (E.J.H.J.); mj492@cam.ac.uk (M.H.J.); 4 Department of Psychology, Institute of Psychiatry, Psychology & Neuroscience, King’s College London, London SE5 8AF, UK; tessel.bazelmans@kcl.ac.uk (T.B.); greg.pasco@kcl.ac.uk (G.P.); tony.charman@kcl.ac.uk (T.C.); 5 Department of Psychology, University of Cambridge, Cambridge CB2 3EB, UK

**Keywords:** ADHD, infant, attention, activity level

## Abstract

Mapping infant neurocognitive differences that precede later ADHD-related behaviours is critical for designing early interventions. In this study, we investigated (1) group differences in a battery of measures assessing aspects of attention and activity level in infants with and without a family history of ADHD or related conditions (ASD), and (2) longitudinal associations between the infant measures and preschool ADHD traits at 3 years. Participants (*N* = 151) were infants with or without an elevated likelihood for ADHD (due to a family history of ADHD and/or ASD). A multi-method assessment protocol was used to assess infant attention and activity level at 10 months of age that included behavioural, cognitive, physiological and neural measures. Preschool ADHD traits were measured at 3 years of age using the Child Behaviour Checklist (CBCL) and the Child Behaviour Questionnaire (CBQ). Across a broad range of measures, we found no significant group differences in attention or activity level at 10 months between infants with and without a family history of ADHD or ASD. However, parent and observer ratings of infant activity level at 10 months were positively associated with later preschool ADHD traits at 3 years. Observable behavioural differences in activity level (but not attention) may be apparent from infancy in children who later develop elevated preschool ADHD traits.

## 1. Introduction

Attention Deficit Hyperactivity Disorder (ADHD) is a neurodevelopmental condition estimated to affect 3–5% of the population [1,2] across high, middle and low-income countries [2,3]. Characterised by symptoms of inattention, hyperactivity and impulsivity [4], ADHD can negatively impact life expectancy and quality of life [5]. ADHD often co-occurs with a variety of genetic [6], psychiatric [7,8,9] and neurodevelopmental conditions [10,11,12], particularly Autism Spectrum Disorder (ASD). Estimates of the proportion of children with ASD who also show clinically significant ADHD symptoms range from 30% to 80% [10], and within this group ADHD symptoms are associated with lower adaptive functioning and quality of life [13,14].

A number of possible causal pathways for ADHD have been proposed [15], all of which involve a variety of genetic and environmental factors that act early in development [16]. Although ADHD is typically diagnosed in middle childhood [17], longitudinal studies indicate continuity in the developmental pathway from the preschool years [18,19]. Later in development, the ADHD phenotype may be further compounded through atypical interactions with the child’s environment, and resulting compensatory processes, or cascading effects [20]. Therefore, once ADHD is diagnosed in childhood (often in co-occurrence with other conditions) it can be difficult to separate the contribution of individual factors from the emergence of ADHD symptoms. Understanding the early developmental roots of ADHD is thus critical to unpicking its aetiology.

Greater insight into the early development of ADHD is also required to develop early intervention approaches [21,22], with the aim of improving later outcomes for individuals with a greater chance of developing the condition. Indeed, early symptoms may impact later functioning: ADHD symptoms at age 3 are associated with poorer mental health outcomes in adulthood [23,24]. Designing early interventions requires an understanding of the causal pathways to ADHD from its earliest manifestations [25]. In particular, being able to detect neurocognitive and behavioural markers of ADHD prior to the onset of symptoms could help to identify individuals who may benefit from additional support earlier, and could provide useful outcome markers for early interventions [20,26].

Two domains that are conceptually related to the core features of ADHD are focused attention and activity level. Across a number of studies, increased activity levels during the preschool years have been shown to associate with both concurrent [27] and later [28,29] ADHD symptoms. Focused attention, a behavioural phase of attention that involves engagement behaviour with a stimulus and the active intake of information [30], has also been shown to relate to ADHD symptoms. More specifically, shorter epochs of infant focused attention in both naturalistic [31] and screen-based [32] contexts have been shown to relate to preschool inattention and hyperactivity. Though diagnostic status may shift over development [33], preschoolers with ADHD traits can experience significant difficulties [34] that may also impact on family quality of life [35,36]. In order to improve early identification and support, it is therefore important to investigate the developmental trajectories of attention and activity level before such challenges emerge in the preschool years.

One way to identify early differences in cognition and brain functioning associated with ADHD is to study infants who have an elevated likelihood of developing later ADHD because they have a family member with ADHD or a related condition (such as ASD). This study design leverages the fact that ADHD is highly heritable, with increased prevalence in individuals with first degree relatives with ADHD [37,38] or ASD [39,40]. Several research groups have launched prospective longitudinal studies to understand early manifestations of ADHD liability in infants with a family history of ADHD [41,42,43,44] or ASD [45]. This body of work has often concentrated on two domains of early functioning that, as discussed above, are linked to ADHD symptoms: focused attention and elevated activity level. From as young as 7 months, infants with a family history of ADHD have been reported to demonstrate lower levels of focused attention, and higher levels of activity (in comparison to infants without a family history of ADHD) as measured by parent-report questionnaires and observations of infant attention during toy play [41,42,46]. Population-based studies have also indicated that behavioural ratings of activity level and attention in the first year of life are associated with later ADHD symptoms [28,47,48]. However, very little is known about the neurocognitive systems in infancy that underlie later behaviours related to the ADHD phenotype.

Focused attention, in particular, is challenging to assess during infancy. Focused attention undergoes substantial development over the first year of life [49], and its non-linear developmental trajectory can make it difficult to disentangle the role of attentional focus from processing speed, and other cognitive processes [50,51]. Furthermore, different measures of infant attention such as fine-grained experimental measures (such as eye tracking) versus observational measures of attention in naturalistic contexts, often show low convergence when measured at the same time point [52]. It is also possible that differences in neurocognitive markers of focused attention and/or activity may emerge prior to changes in global behaviour. Therefore, in this study, we opted to use a multi-method assessment protocol, involving a range of both neurocognitive and behavioural measures to assess infant attention and activity. Our measures of infant attention and activity were pre-registered prior to analysis (see https://osf.io/kyc46, registered on 28 July 2017).

At the global behavioural level, in line with previous prospective studies of infants with a family history of ADHD [41,46], we assessed group differences in infant focused attention and activity level using global ratings of behaviour from parents and observers. Parent and observer ratings can be seen as complementary. Parents are able to provide insights into the natural variation in particular behaviours across different contexts [53], whilst observers of a semi-standardized laboratory session provide an overview of behaviour during tasks specifically selected to elicit variation in the behaviours of interest (in this case focused attention and activity). In preschoolers, clinicians’ ratings of preschool attention and activity are more predictive of later ADHD than parent ratings alone [54], highlighting the value of a multi-informant approach for global ratings of behaviour. Using parent-report questionnaires, previous studies have found higher levels of activity and lower levels of attention in infants with a family history of ADHD (compared to infants with no family history of ADHD) from as early as 7 months [41]. Recent literature suggests that parent-report of infant activity level shows specificity to later ADHD symptoms, rather than symptoms of co-occurring conditions such as autism and anxiety [55]. In a recent study, children with a family history of ASD who later received a diagnosis of ADHD (but not ASD) showed elevated observer ratings of inattentive and hyperactive/impulsive behavior at 18 months, although parental concerns did not differ from those of typically developing infants until at least 24 months [45].

Global measures of behaviour are comprehensive but may miss more subtle changes that may occur early in the development of ADHD. Semi-structured play-based assessments afford an opportunity for more fine-grained analysis of attention-related behaviors than parent or observer report. Behavioural-cognitive measures of infant focused attention, such as the duration of time the infant spends manipulating objects whilst looking at them, show some continuity to later focused attention in the toddler and preschool years [51]. Amongst infants born pre-term, lower spans of focused attention during toy play at 7 months are associated with higher ADHD traits at age 4–5 years [56]. In a previous prospective study, 7 month old infants with a family history of ADHD showed less focused attention during toy play compared to infants without a family history of ADHD [42]. Thus, in the present study, we examined two indices of infant attention (longest epoch, and total duration) during naturalistic toy play with a set of blocks.

Whilst observational measures of behaviour provide insight into subtle differences in attention in a natural setting, measures of visual attention taken through eye-tracking can provide more fine-grained dissection of the cognitive processes underlying behaviour change. In the present study, two eye-tracking measures of infant visual attention were used to capture focused attention. In developmental research, “peak look duration” (the longest look during passive viewing of a static stimulus in an eye-tracking paradigm; [57]) is often thought to reflect focused attention. Indeed, infant peak look duration has been associated with elevated polygenic scores for ADHD [58], later ADHD traits in mid-childhood [58] and toddler executive functioning [59]. A second measure is greater variability in reaction times during a paradigm requiring attention. Reaction time variability (RTV) is increased in some children with ADHD, and is often thought to reflect lapses in attention [60,61] (though other theoretical models of RTV in ADHD have also been proposed [62]). Reaction time variability has been highlighted as a possible endophenotype for ADHD [63] and a significant proportion of the genetic influences shared between inattention and autistic-like traits can be accounted for by RTV [64]. In this study, we measured reaction time variability (RTV) during a visual attention shifting task [65].

Epochs of looking can be associated with different levels of engagement with the stimulus being fixated; sometimes, infants may stare “blankly” at an object and this may not represent truly focused attention. Examining physiological arousal can provide further insight into periods of infant focused attention [66]. Moreover, arousal dysregulation during tasks that require attention has often been reported as a feature of ADHD [67]. At a physiological level, increased behavioural stillness [30] and lowered heart rate [66,68] are expected during periods of infant focused attention. In this study, we used the amount of head motion observed during passive viewing of static stimuli and average heart rate during naturalistic toy play as physiological measures of attention.

Finally, we also examined infant attention at the neural level. Specifically, we focused on EEG theta power. Infant theta oscillations have been associated with infant attention in a number of studies [66,69,70,71,72], and maternal ADHD symptoms are related to higher mean infant theta power [73]. In this study we investigated both mean frontal theta and dynamic changes in frontal theta. Dynamic changes in theta power in response to a novel stimulus are thought to reflect attention in the service of learning and memory [71], and may provide an even more sensitive measure of infant attention than using mean power alone.

The primary aim of our study was to investigate whether a family history of ADHD or related conditions (ASD) was associated with differences in early attention and activity level at 10 months of age. We focused on 10 months of age, as the latter part of the first year of life is proposed to be a critical period of development for attentional focus [51]. This may represent a particular sensitive period, both in terms of the detection of possible group differences, but also potentially as an optimal time period in which to implement early interventions targeting emerging atypicality [22]. In summary, our pre-registered hypotheses (https://osf.io/kyc46, registered on 28 July 2017) were that in comparison to infants with no family history of ADHD, infants with a family history of ADHD would show: higher parent and observer reported scores of activity level, lower behavioural attention during toy play, shorter peak look durations and greater reaction time variability during eye-tracking, higher mean heart rate and greater head motion during experimental paradigms designed to elicit attention, increased mean frontal theta and decreased change in frontal theta power in response to novel videos.

The secondary aim of our study was to investigate longitudinal associations between infant activity level and attention at 10 months of age and ADHD-related behaviours at 3 years (including preschool ADHD traits, and temperament dimensions conceptually related to ADHD). These analyses were hypothesis-driven but not pre-registered. We predicted that parent and observer reported activity level, reaction time variability, heart rate during naturalistic play, head motion and mean frontal theta would be positively associated with preschool ADHD traits. Further, we predicted that behavioural attention (parent and observer report), peak look duration (during naturalistic play and an eye-tracking task) and change in frontal theta would be negatively associated with ADHD-related behaviours.

Finally, in exploratory analysis, we also examined the extent to which our pre-registered measures of infant activity and attention converged. This exploratory analysis has value not only in interpreting the results of our own study, but also in contributing more broadly to the field in terms of the measurement of attention in infancy.

## 2. Methods

### 2.1. Participants

Participants were recruited as part of the Studying Autism and ADHD Risks (STAARS) project. STAARS is an ongoing longitudinal study following the development of infants who either have a first-degree relative with a diagnosis or probable research diagnosis of ADHD, and/or a diagnosis of ASD (and thus are at elevated likelihood of ADHD), or who have no family history of ADHD and ASD (and thus are at typical likelihood of ADHD). We had originally preregistered analyses to be conducted with infants at typical likelihood of ADHD and infants with a family history of ADHD only; however, in line with current moves towards transdiagnostic approaches in studying the early development of neurodevelopmental conditions [43,74] we subsequently expanded our analysis to include an additional cohort of infants with a family history of ASD. Infants with a family history of ASD are also at elevated likelihood of ADHD [10], and for autistic individuals, ADHD symptoms can impact outcomes [13,14]. Therefore, in the present study we examined the effect of family history of ASD, family history of ADHD, and their interaction. For transparency, in SM1 we also present results of the analyses excluding infants with a family history of ASD (and no family history of ADHD), as per the pre-registration.

Families were invited to visit the laboratory at the Centre for Brain and Cognitive Development (CBCD) when their infant reached 5, 10 and 14 months of age, and King’s College London when their infant reached ~3 years of age. Infants who met any of the following criteria based on parent report at screening were excluded: (1) serious medical or developmental conditions, (2) significant uncorrected vision or hearing problems, (3) significant prematurity (less than 36 weeks’ gestation), (4) genetic conditions such as Down’s syndrome or Fragile X syndrome.

The sample for the present study comes from the first wave of participants, which comprises infants who attended their first visit to the laboratory for the project prior to June 2017 (*N* = 151); see SM2 for exclusions prior to analysis. The present study uses data from the 10-month time point.

Demographic characteristics for the study sample at 10 months and 3 years are shown in Table 1. All parents gave informed consent for their infant to participate in the study. Infants were given a certificate and t-shirt after each visit to the laboratory. The study was approved by the National Research Ethics Service: London Central (13/LO/0751). Of note, a small subset of the infants included in this study received a 9-week attention training programme following their 10-month lab visit (*N* = 10), as part of a randomised controlled trial [22].

### 2.2. Classification of Family History Status

Information about first-degree relative diagnostic status was ascertained through a number of methods. Before families enrolled in the study, a telephone screening form was used to determine the presence of an existing community clinical diagnosis of ASD (“FH-ASD”) or ADHD (“FH-ADHD”) in a first-degree relative (parent or older sibling). This was confirmed by interview with parents at a subsequent visit to the lab. A proportion of children/parents had suspected ADHD (*N* = 9), but this had not yet been confirmed by clinical services. For those who reported suspected ADHD, screening questionnaires were used to examine the probable existence of ADHD (SM4). Inclusion decisions were reviewed by the project management team. Our categorisation protocol is similar to that adopted by other labs and studies using the prospective longitudinal study model in infants at elevated likelihood of ADHD and ASD (e.g., [46]). We defined “FH-No ADHD/ASD” as infants who had at least one older sibling and no first-degree relatives with a diagnosis of ASD or ADHD.

For analysis, each infant in the study was assigned a classification for family history of ASD and ADHD separately. A rating of 1 for ASD family history indicates a diagnosis of ASD in a parent or older sibling; a rating of 1 for ADHD family history indicates a diagnosis or probable research diagnosis of ADHD in a parent or older sibling; and a rating of 0 for either category indicates no confirmed presence of the relevant condition. This approach allowed us to separately test the effect of family history of ASD, family history of ADHD, and their interaction.

### 2.3. Measures of Infant Attention and Activity Level

We assessed infant activity level and attention across six modalities, as described below. For each modality, all infants with valid data for that modality were included, regardless of whether they had data for other modalities. For data exclusion thresholds see https://osf.io/kyc46 (registered on 28 July 2017). Missing data per measure is described in SM5.

 

(1) Global ratings of activity level

**Parent-report.** Parents were asked to complete the Infant Behavioural Questionnaire-Revised (IBQ-R) shortened version [75]. The IBQ-R asks parents to rate how often their child performed a range of behaviours during the last week on a 1 (never) to 7 (always) scale. We selected the Activity Level scale, which includes items such as “When placed in an infant seat or car seat, how often did the baby wave arms and kick”, and “When placed on his/her back, how often did the baby squirm and/or turn their body”. Previous reports with typically developing infants have found moderate inter-parent agreement and homotypic continuity over spans of up to 7 months for the Activity Level scale of the IBQ-R [75]. In our sample, Cronbach’s alpha for the Activity Level scale was 0.73, indicating good internal consistency.

**Observer report.** Each 10-month visit at the CBCD lasted approximately 5 h, generally spanning both morning and afternoon, led by two researchers. Following each laboratory visit, the two researchers discussed and agreed a consensus code for the infant’s attentiveness and activity level across the whole 10-month visit (ranging from 1–7). For activity level this ranged from 1 (low activity level) to 7 (high activity level). To ensure consistency across infants, at least 1 of these researchers was a “Core consensus coder” (1 of 2 researchers who contributed to every 10-month consensus code). Core consensus coders had PhD level training.

 

(2) Global ratings of attention

**Parent-report.** Parents were asked to complete the IBQ-R, as described above. We selected the Duration of Orienting scale. This subscale measures infants’ attention and/or interaction with a single object for extended periods, and includes items such as “How often during the last week did the baby play with one toy or object for 5–10 minutes”, and “How often during the last week did the baby look at pictures in books and/or magazines for 5 minutes or longer at a time”. Previous reports with typically developing infants have found moderate inter-parent agreement and homotypic continuity over spans of up to 7 months for the Duration of Orienting Level scale of the IBQ-R [75]. In our sample, Cronbach’s alpha for the Duration of Orienting scale was 0.76, indicating good internal consistency.

**Observer report.** As described above, consensus codes of infant attentiveness across the whole 10-month visit was rated by two researchers. For attentiveness this ranged from 1 (low attentiveness) to 7 (high attentiveness).

 

(3) Behavioural measures of attention

**Laboratory Temperament Assessment Battery (LabTAB).** The task orientation episode from the Laboratory Temperament Assessment Battery, LabTAB [76] was used to measure infant attention during toy play. Infants were seated in a high chair, or on their parent/caregiver’s lap, and given a set of toy blocks to play with for 3 minutes. Infant behaviour was video recorded to capture the infant’s face, hands and gaze. The time the infant spent looking at and manipulating the blocks was coded offline using Mangold INTERACT version 15. All variables were coded continuously at 25 frames per second (full coding scheme; see SM6). As a measure of attention, “Active Attention”, was operationalised as the time that infants spent looking at the blocks at the same time as manipulating the blocks. The key variables extracted for this study were: (1) the total duration of Active Attention (“Total Active Attention”) and (2) the maximum duration of any individual epoch of Active Attention (“Peak Active Attention”). Data was coded by five observers blind to family history classification. Before coding, all observers independently coded subsets of 10–12 videos for inter-rater reliability (Intraclass Correlation Coefficient for “Active Attention”, two way mixed, single measures, absolute agreement > 0.7). Visual inspection of the data indicated that the “Peak Active Attention” variable was non-normally distributed; therefore, this was log transformed prior to analysis.

 

(4) Eye tracking measures of attention

Two eye-tracking tasks were administered as part of a larger battery (~30 minutes) of eye tracking tasks, using a Tobii TX-300 eye tracker (Tobii AB, Stockholm, Sweden) sampling at 120 Hz. The screen had a diagonal size of 23” (58.4 cm × 28.6 cm, 52.0° × 26.8° @ 60 cm), a native resolution of 1920 × 1080 pixels and an aspect ratio of 16:9. Stimuli were presented on Apple (Apple Inc., Cupertino, CA, USA) Macbook Pro computers, using our custom-written stimulus presentation framework (Task Engine, sites.google.com/site/taskenginedoc/), running in Matlab R2020b (The MathWorks Inc., Natick, MA, USA) using Psychtoolbox 3 [77,78].

**Face pop-out task [79].** Infants were presented with a series of six annular visual arrays each composed of five objects in different locations on the screen. Each array contained: a face with direct gaze; a visual “noise” image generated from the same face presented within the array by randomising the phase spectra of the face whilst keeping the amplitude and colour spectra constant to act as a control for the low-level visual properties of the face stimuli [80]; and an image of a mobile phone, a bird and a car. The exemplar faces and objects varied between trials. Each array was presented for 10 seconds and counter-balanced for the location of the face in the array. The stimulus array was 43.8 cm × 28.6 cm (39.0° × 26.8° @ 60 cm). The key task variable—peak look duration—was the maximum look duration to any individual stimulus (object or face) for each trial, averaged across trials. Trials were excluded if they were shorter than 5 seconds, or had less than 25% valid data. See https://osf.io/kyc46 (Appendix 2) (registered on 28 July 2017) for a full description of analysis steps and data processing pipelines for this task. Previous work has shown that infants’ average duration of fixations shows high test-retest reliability [81] and that automated assessment of fixation duration is not significantly impacted by group differences in data quality [82].

**Gap-overlap task [65].** For reaction time variability, our selected variable was the coefficient of variation for saccadic reaction times in the overlap condition of the gap-overlap task. All stimuli were presented at a size of 3 cm × 3 cm (2.86° × 2.86° at 60 cm viewing distance). In the overlap condition, each trial started with the onset of a central stimulus (CS), a cartoon image of an analogue clock accompanied by an alerting sound. This pulsed on screen at 3 Hz between 3 cm–5 cm (2.86°–4.77°) until fixated by the participant. The CS then rotated at 500° per second for a random 500–700 ms ISI. After 200 ms, a peripheral stimulus (PS) was presented, while the CS remained on screen for the duration of the rest of the trial. The PS was a cartoon cloud that appeared on either the left or the right side of the screen and was accompanied by a sound, 3 cm (2.86°) from the edge, rotating at 500° per second until fixated by the participant. A reward stimulus was then presented at the location of the PS for 1000 ms. The reward was a randomly chosen cartoon image of either a star, a sun, a dog, cat, pig, tiger or tortoise, accompanied by a sound. To encourage engagement the reward stimulus was animated to either spin on the spot, spin and shrink, or to pulse. Data were analysed offline. Each trial was inspected automatically to determine trial validity and calculate a saccadic reaction time (SRT) to shift attention from the CS to the PS, relative to PS onset. A trial was valid if the following conditions were met: (1) gaze fell on the CS; (2) no gaps of missing data longer than 200 ms were present during the CS period (before PS onset); (3) there was at least one sample of gaze on the CS within 50 ms either side of PS onset; (4) no gaps of missing data longer than 100 ms were present during the PS period (between PS onset and reward onset); (5) SRT was longer than 150 ms and shorter than 1200 ms; (6) gaze did not go in the opposite direction to the side of the PS; (7) gaze did not enter the PS Area Of Interest (AOI) after engagement with the CS but before PS onset. Trials that failed any of the above criteria were invalidated and removed from further analysis. See https://osf.io/kyc46 (Appendix 3) for a full description of analysis steps and data processing pipelines for this task. Previous work indicates that SRTs for the baseline condition of the Gap-overlap task show moderate test-retest reliability across a 1-week period amongst typically developing 10-month-old infants [83].

 

(5) Physiological measures of attention

**Heart rate.** Average heart rate during the LabTAB task orientation episode (as described above) was used as a physiological measure of attention and arousal during toy play. Heart rate data was collected using the RSPEC BioNomadix (BIOPAC Systems, Inc, Goleta, CA, USA), attached to a MP150 amplifier, sampled at either 100 or 1000 Hz. Three electrodes were placed in a lead-II position on the child’s back. The transmitter was placed in the pocket of a one-piece baby suit designed for the study. AcqKnowledge (version 4.4, BIOPAC Systems, Inc., Goleta, CA, USA) was used for data acquisition. R-peaks were visually identified in AcqKnowledge and exported to Matlab. Missing R-peaks were corrected up to 3 consecutively missing beats by dividing the interbeat interval. Data was analysed in 30-second epochs, which were marked invalid if four or more consecutive beats were missing. The average of all valid epochs is used as the participant’s average heart rate for the task. Any 30-second epochs with more than 10% of peaks missing were excluded. See SM7 for a full description of analysis steps and data processing pipelines for this measure. Previous work indicates moderate-to-good test-retest reliability of average heart rate for both autistic and typically developing toddlers [84].

**Head motion.** Head motion was analysed during the face pop-out eye tracking task. The face pop-out task was selected as it is distributed throughout the eye tracking battery, involves passive viewing, and does not involve exogenous “attention getters”. During the face pop-out eye tracking task, head motion was derived from the 3D coordinates of each eye, as reported by the eye tracker and down-sampled to 30 Hz. Distance is the first derivative (the Euclidean distance between consecutive samples), and velocity is the second derivative. To normalise for quantity of valid, binocular samples (i.e., amount of clean data during which the infant attended to the screen) we used mean velocity as the derived variable to indicate amount of head motion. After smoothing, sample-by-sample velocity was derived and the mean velocity calculated for each trial. See SM8 for a full description of analysis steps and data processing pipelines for this measure.

 

(6) Neural measures of attention

**EEG.** EEG was recorded while infants were presented with two 1-minute videos (one social, one non-social) each presented twice during the EEG session. In the social video, infants were presented with two women (one at a time) telling nursery rhymes with gestures. In the non-social condition, infants were present with child appropriate toys moving on the screen (such as coloured balls moving down a chute), with no social content. The order of the videos was counterbalanced across infants. EEG data was acquired using a 128 electrode Hydrocel Geodesic Sensor Net 1.0, recorded online with reference to the vertex, digitized at 500 Hz, amplification at 1000×. Using Netstation 4 (version 4.5.6) (EGI, Eugene, OR, USA) band pass filtering at 0.1 to 100 Hz was applied. The EEG data was segmented into 1 second segments. Segments where infants were not looking at the video were removed from further analysis. Artefact detection was completed using hand editing (AG, EJHJ, LF). Data was referenced to the average reference, and the resulting segmented data was imported into Matlab. Within Matlab, segments were detrended and subjected to a Fast Fourier Transform (FFT). Power values were averaged across artefact-free segments and electrodes, within a priori of topographical groups. The topographical group of interest for this study were the frontal electrodes (SM9).

Theta power values were calculated for two time intervals—Time 1: the first half of the video (0–30 seconds), and Time 2: the second half of the video (30–60 seconds)—for each 1 minute video. Natural logs were calculated to reduce skew. Logged power values were averaged across the theta (3 to 6 Hz) frequency range. Primary analyses collapsed across social and non-social conditions. The following formula was used to calculate change in frontal theta power averaged across the first presentation of the social and non-social video (Power30–60 s − Power0–30 s)/Power0–30 s × 100%. Only the first video repetition was used, because the second repetition would be contaminated by familiarity [71]. For frontal theta change, at least 10 artifact free segments were required for both the first and second half of the first video. Mean theta power was calculated as absolute frontal theta power in the first 30 seconds of each video, averaged across the four videos. For mean frontal theta, at least 10 artifact free segments were required for the first half of at least one video repetition.

### 2.4. Measures of Preschool ADHD Traits at 3 Years

**Child Behaviour Checklist (CBCL).** We used the DSM-oriented ADHD subscale of the CBCL (1 ½–5) to measure preschool ADHD traits at 3 years of age [85]. This 6-item subscale asks parents to rate how well a statement describes their child’s behaviour as observed within the last 2 months on a 3-point scale from 0 (“Not True”) to 2 (“Very True or Often True”). To be included in analysis, a minimum of 5 items was required. This version of the CBCL has shown high discriminative ability to identify ADHD diagnoses in large samples of preschoolers [86].

**Child Behaviour Questionnaire (CBQ).** We used the CBQ short version to measure broader temperament dimensions related to the preschool ADHD phenotype at 3 years of age [87]. The CBQ short version contains 94 items that asks the parent/caregiver to rate how often their child performs a range of behaviours on a 1 (never) to 7 (always) scale. We selected the Impulsivity, Attentional Focusing, Activity Level and Inhibitory Control scales for analyses. For each scale, participants were included in the analysis if they had at least 60% of the items for that scale. Cronbach’s alpha for each subscale was as follows: Activity Level (0.71), Attentional Focusing (0.69), Impulsivity (0.68), Inhibitory Control (0.64).

### 2.5. Analytic Strategy

**Effect of family history of neurodevelopmental disorders at 10 months.**

The effect of family history on each of the six modalities described above was evaluated in a series of multivariate analyses of variance (MANOVAs), using the measures listed above as dependent variables such that there were two dependent variables for each MANOVA (e.g., for the global activity level MANOVA the dependent variables were IBQ-R Activity Level scores, and Observer report of activity level consensus scores). Family history classification (FH-ADHD, FH-ASD and the interaction between FH-ADHD and FH-ASD) were used as fixed factors in each MANOVA. For each modality, all infants with valid data for all measures in that modality were included, regardless of whether they had data for other modalities. Missing data per measure is described in SM5.

Partial eta squared values indicate the proportion of variance associated with membership of that group (e.g., FH-ADHD) after controlling for other factors in the model (e.g., FH-ASD and the interaction effect of FH-ASD and FH-ADHD). As this is considered a biased estimate [88], we also present partial omega squared values—computed using https://osf.io/kzsyg/ (accessed on 29 March 2021) and set to 0 for negative values—and interpret both values using the heuristics suggested by Cohen (1988) [89] whereby an effect size of 0.0099 can be considered small, 0.0588 medium, and 0.1379 a large effect. The results from the full model are presented below (Table 2). For the pre-registered analyses that do not consider FH-ASD (as per https://osf.io/kyc46, registered on 28 July 2017), see SM1; results are substantively the same. We also conducted follow-up MANCOVAs controlling for age and sex (using the same dependent variables and fixed factors as in the MANOVA analyses described above, with infant age in days, and sex, as covariates). Both age and sex have been related to measures of activity level and/or attention during the first year of life; [57,90,91], and so it was pertinent to understand how these variables may have affected results. Primary interpretations were made using the results from the MANOVA, due to greater power.

**Longitudinal associations of infant attention and activity level with preschool ADHD traits at 3 years.**

Participant characteristics for the sample retained at 3 years are shown in Table 1. A series of robust regression models were used to test how each of the measures we had selected to assess infant attention and activity level at 10 months of age related to emerging preschool ADHD traits at 3 years of age (across the full cohort of infants). For each regression model, the outcome variable was the DSM-oriented ADHD subscale of the CBCL. Each modality of infant attention and activity was entered as the predictor variable(s): global ratings of activity level from parents and observers (Model 1); global ratings of attention from parents and observers (Model 2); behavioural measures of active attention (Model 3); eye tracking measures of attention (Model 4); physiological measures of attention (Model 5); and neural measures of attention (Model 6). For models which significantly predicted preschool ADHD traits at 3 years, we further investigated the relationship of these infant measures with broader temperament dimensions that are conceptually related to the ADHD phenotype, as a method of triangulation. Specifically, we selected the impulsivity, activity level, inhibitory control, and attentional focusing subscales of the Child Behaviour Questionnaire (CBQ) as outcome variables at 3 years.

**Associations between measures of attention and activity level at 10 months.**

We used Spearman’s correlations to investigate the association between our measures of attention and activity level at 10 months. Given the exploratory nature of this analysis, *p*-values were not corrected for multiple testing.

## 3. Results

### 3.1. Effect of Family History of Neurodevelopmental Disorders at 10 Months

**Figure 1:**
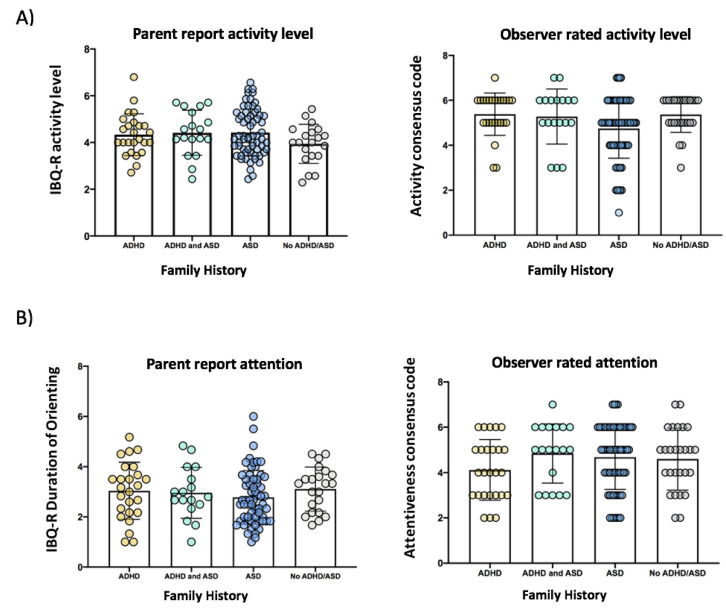

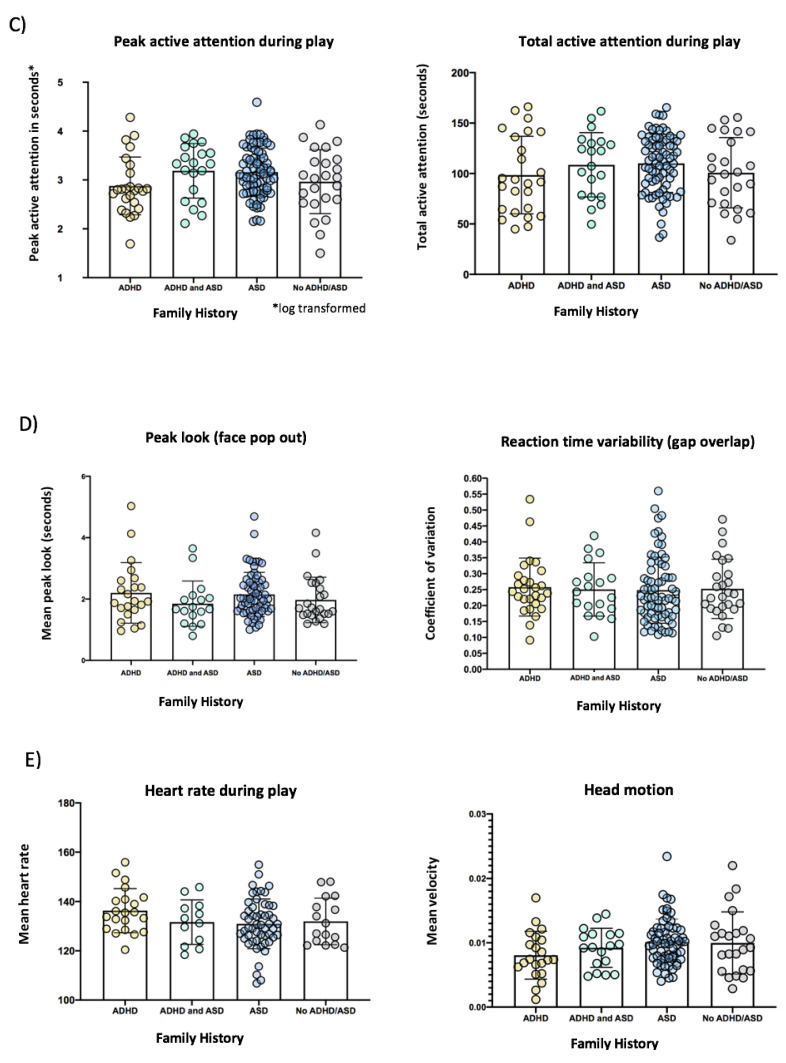

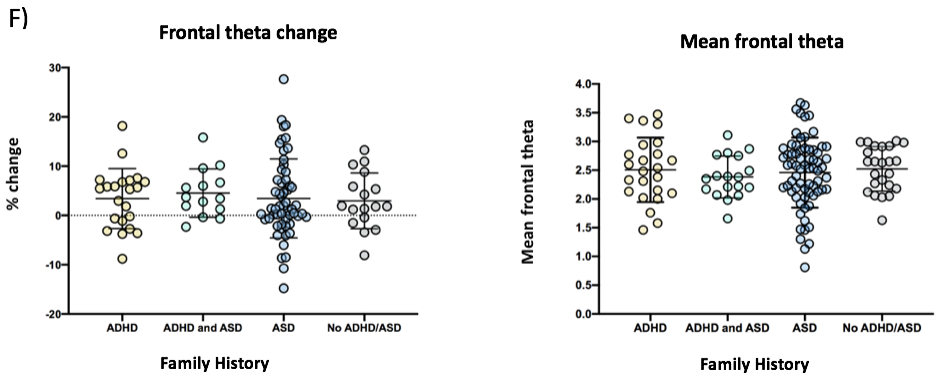
Bar plots with scatter overlay for the six modalities of infant attention and activity level at 10-months including: (**A**) global ratings of activity level, (**B**) global ratings of attention, (**C**) behavioural measures of active attention, (**D**) eye tracking measures of attention, (**E**) physiological measures of attention, and (**F**) EEG measures of attention, for infants with a family history of ADHD only (“ADHD” shown in yellow), infants with a family history of both ADHD and ASD (“ADHD and ASD” shown in green), infants with a family history of ASD only (“ASD” shown in blue) and infants without a family history of either condition (“No ADHD/ASD” shown in grey). Error bars are +/−1 SD.

In our primary analyses, the MANOVA revealed no significant effect of family history of ADHD or ASD on any of the behavioural or neurocognitive measures of activity level and attention at 10 months of age, see Figure 1, and Table 2. Follow-up MANCOVA controlling for infant sex and age also revealed no significant effects.

### 3.2. Associations between Infant Measures of Attention and Activity Level at 10 Months

**Figure 2:**
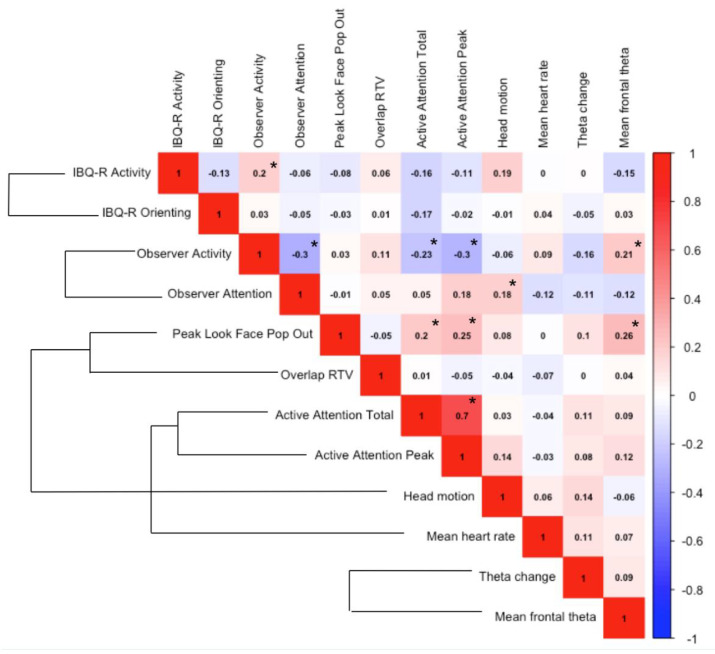
Correlation matrix heatmap of measures of infant attention and activity level for the full sample at 10 months. External brackets indicate that the two measures come from the same testing domain (e.g., the same EEG or eye-tracking testing session). Spearman r values are shown. Significant correlations (* *p* < 0.05) are denoted by an asterisk. Given the exploratory nature of this analysis, *p*-values were not corrected for multiple testing.

Informed by the previous literature, our 10-month measures were selected to assess infant activity and attention at both the behavioural and neurocognitive level. Across the full sample of infants, we used Spearman’s correlations to investigate the association between our measures of attention and activity level at 10 months of age, see Figure 2 below. There was limited association between measures from different modalities (global, behavioural, eye-tracking, physiological or neural) at 10 months of age. One exception was behavioural attention (Active Attention Total and Active Attention Peak), which showed a weak-to-moderate negative association with a global measure of activity (Observer rating of activity level), and a weak positive association with an eye-tracking measure of attention (Peak Look duration during the Face Pop-Out task). Peak look duration during the Face Pop-Out task also showed a weak positive association with one of the neural measures (mean frontal theta).

### 3.3. Longitudinal Associations of Infant Attention and Activity Level with Preschool ADHD Traits at 3 Years

**Figure 3:**
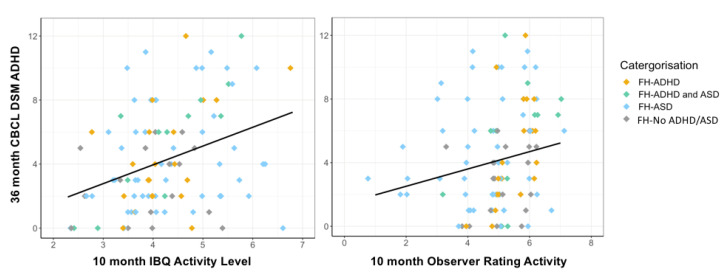
Infant activity level and 3-year preschool ADHD traits. Scatterplot for the full cohort with regression line showing 10-month activity level (left: IBQ-R Activity Level subscale, and right: observer ratings of activity level) on the x-axis, and the DSM-oriented ADHD subscale of the Child Behaviour Checklist (CBCL) at age 3 years on the y-axis, colour coded by family history group, with jitter due to discrete scales.

Descriptive statistics for the 3-year outcome measures are shown below in Table 3 (for CBCL and CBQ scores split by family history status, see SM10). At age 3, children with a family history of ADHD scored higher on the DSM-oriented ADHD subscale of the CBCL compared to children without a family history of ADHD or ASD, F (1, 54) = 5.17, *p* = 0.027, partial η^2^ = 0.87. Consistent with our prediction, global measures of activity level at age 10 months were positively associated with ADHD-related behaviours at age 3 years. For the full cohort, robust regression showed that global ratings of activity level at 10 months of age (Model 1) was a significant predictor of CBCL ADHD traits at 3 years, F (2, 93) = 9.70, *p* < 0.001, with parent-reported activity level (IBQ-R) contributing significantly to the model (*B* = 1.26, SE = 0.34, *p* < 0.001), and observer ratings of activity level more weakly contributing (*B* = 0.49, SE = 0.27, *p* = 0.067), Figure 3. Contrary to our prediction, we found no significant positive association between measures of attention at 10 months and ADHD-related behaviours at age 3 years. For the full cohort, robust regression showed that CBCL ADHD traits at 3 years was not predicted by global ratings of attention, F (2, 94) = 1.13, *p* = 0.33; behavioural measures of attention, F (2, 107) = 0.30, *p* = 0.74; eye tracking measures of attention, F (2, 92) = 0.84, *p* = 0.44; physiological measures of attention, F (2, 61) = 1.22, *p* = 0.30; or neural measures of attention, F (2, 80) = 0.09, *p* = 0.91.

For the significant model (Model 1), we further investigated the relationship of infant activity with broader temperament dimensions conceptually related to the ADHD phenotype at 3 years. Four separate robust regression models were conducted with each selected CBQ subscale as the outcome variable (Activity Level, Attentional Focusing, Impulsivity, and Inhibitory Control). A corrected *p*-value of 0.0125 was applied. Infant global activity ratings (from parents and observers) were entered as the predictor variables in all models. Robust regression showed that global ratings of activity level at 10 months was a significant predictor of Activity Level, F (2, 91) = 5.18, *p* = 0.007, Impulsivity, F (2, 90) = 3.70, *p* = 0.028, and Inhibitory Control, F (2, 90) = 9.59, *p* < 0.001, at 3 years, though the Impulsivity model did not survive correction for multiple testing. For Activity Level, parent-reported activity level contributed significantly to the model (*B* = 0.31, SE = 0.10, *p* = 0.003), but not observer ratings of activity level (*B* = 0.06, SE = 0.08, *p* = 0.46). For Inhibitory Control, both parent-reported activity level (*B* = −0.30, SE = 0.10, *p* = 0.004) and observer-reported activity level (*B* = −0.21, SE = 0.08, *p* = 0.006) contributed to the model. Similar results were found for Impulsivity but with weaker effects (observer ratings, *B* = 0.13, SE = 0.07, *p* = 0.059; parent ratings *B* = 0.15, SE = 0.09, *p* = 0.104). Global ratings of activity level at 10 months of age did not predict Attentional Focusing at 3 years, F (2, 91) = 1.65, *p* = 0.20. For scatterplots of infant activity level and each CBQ outcome, see SM11.

## 4. Discussion

ADHD is a heritable neurodevelopmental condition that is typically diagnosed in middle childhood, but which is likely influenced by genes that act prenatally, as well as early environmental factors [92]. Identification of early developmental pathways to the full ADHD phenotype is critical for understanding its aetiology, and for establishing possible targets for early interventions. The present study used a multi-modal battery of behavioural and neurocognitive measures to examine two key candidate early phenotypes that are conceptually related to ADHD: attention and activity level. Contrary to our predictions and to previous reports [41,42], infants with a family history of ADHD (“FH-ADHD”), and/or ASD (“FH-ASD”) did not show significant differences compared to infants without a family history of neurodevelopmental disorders in any of our selected measures of attention or activity level at 10-months of age. However, parent and observer reports of infant activity level at 10 months of age were significantly associated with ADHD-related behaviours at 3 years; similar associations were not seen with measures of infant attention. Taken together, these results suggest that heightened activity levels may be a precursor of later ADHD traits.

### 4.1. Effect of Family History of Neurodevelopmental Disorders at 10 Months

The lack of significant differences that we observed between infants with and without a family history of ADHD/ASD at 10 months in this study contrasts with the small body of existing literature that has found alterations in attention and activity level in infants at elevated likelihood of ADHD within the first year of life [41,42]. Using parent-report and observations during toy-play, previous studies have reported lower levels of infant attention, and higher levels of infant activity associated with a family history of ADHD, from as early as 7 months of age [41,42,46]. In our study, the negligible-to-small effect sizes observed within each MANOVA indicate that even if our sample size were increased it is unlikely that meaningful significant group differences would be found for most of our measures. When we restricted analyses only to the FH-ADHD group and FH-No ADHD/ASD group we observed a similar pattern of results, indicating that our conclusions were not affected by the presence of children with a family history of ASD (SM1).

There are several possible explanations for why we did not find evidence for early alterations in attention and activity level amongst infants at elevated likelihood of ADHD at 10 months. First, it is possible that we did not select the most appropriate or sensitive measures in this study. It may be that trajectories of infant attention and activity across development are more informative in understanding individual differences in later outcomes [45,59], rather than information collected at a single age point. Indeed, Miller et al. (2018) [45] found that in a sample of infants who were at elevated likelihood of ADHD (because they had a sibling with ASD), infants who later received an ADHD diagnosis did not demonstrate a significant increase in look durations between 3 and 24 months of age during an eye-tracking task, while comparison infants did. Nevertheless, in terms of developing possible screening measures for ADHD, it is also important to develop our understanding of behavioural and neurocognitive markers at single age points.

Another possible explanation of the difference between our findings and that of previous studies is sampling variation. Both autism and ADHD are heterogenous conditions, and many factors including (but not limited to) genetic variation, co-occurring conditions, psychosocial and environmental factors (both prenatally and postnatally) may also influence the development of attention and activity level [16]. Previous work has indicated that amongst infants with FH-ASD, plateaued development of attentional focus is associated with later elevated ADHD traits—but only a minority of FH-ASD infants show this attentional profile [93]. It is possible that the FH-ADHD and FH-ASD infants in our sample may have included fewer individuals with early attentional difficulties than are commonly found amongst this population, or that our FH-No ADHD/ASD group may have had a higher proportion of attentional difficulties than might typically be expected. It is also possible that missing data may have affected our results. In this study, analyses were conducted in line with our pre-registered analysis plan. However, when we conducted univariate analyses on each of the individual variables included in the MANOVA, we observed similar results (SM12) indicating that effects were not driven by missing data for individual measures within each MANOVA. Perhaps infants with elevated activity levels, or different attentional profiles, were more likely to not complete certain experimental measures, or to have been excluded from analyses due to missing trials, or poor data quality. To examine this possibility, we compared missing data for each of the experimental measures between infants in the upper and lower quartile for activity level (IBQ-R activity level) and attention (IBQ-R duration of orienting) respectively. There was no effect of group (high vs low attention or activity) on the presence of missing data (SM13).

Our categorisation approach used in this study is similar to recent studies using a prospective longitudinal design to study infants at elevated likelihood of ADHD (e.g., [46]). However, some previous studies have restricted their family history cohorts to only include parents as ADHD probands (not siblings) [41,42], or to only male samples [41,42]. Such study design decisions may affect ADHD prevalence rates and associated difficulties in the recruited sample. We considered the inclusion of females in our sample as important, given that females with ADHD have often been underrepresented both in research and clinically [94]. However, the transmission of familial likelihood may be different in female, compared to male samples [95]. Furthermore, the profile of ADHD may differ across males and females [94,96]. When we included sex as a covariate in our primary analyses at 10 months, however, results did not differ substantially. Moreover, while at 3 years, males with a family history of ADHD in our cohort showed greater ADHD-related traits on average compared to female infants (based on total CBCL ADHD subscale scores), effect sizes were small and not significant (SM14). We included both siblings and parents as probands in our study, since both have been associated with an increased prevalence of ADHD [37,38,97]. However, it is possible that prevalence rates differ depending on whether it is a parent or sibling who is the proband. Given our relatively small sample size, subgroup analyses (e.g., based on sex, or proband) should be approached with caution. Future large studies, or collaborative networks, should allow for these questions to be probed.

### 4.2. Longitudinal Associations of Infant Attention and Activity Level with Preschool ADHD Traits at 3 Years

We found that activity level at 10 months (as rated by parents and observers) was predictive of preschool ADHD traits at 3 years, measured using the DSM ADHD subscale of the CBCL. These results are consistent with previous research that has associated infant activity level from 7 months of age with later ADHD [41], both in samples at elevated likelihood of ADHD [46,55], and population-based samples [47]. While in the present study we focused on preschool behaviours associated with the ADHD phenotype, there is some evidence in the previous literature that infant activity level shows specificity to ADHD symptoms into mid-childhood, rather than symptoms of co-occurring conditions such as anxiety or ASD [55]. Taken together, these findings suggest that infant activity level is worth pursuing as a potential early marker of ADHD.

In general, parent-ratings of infant activity level were more strongly predictive of 3-year outcomes than observer ratings. Parents are often with their child on a regular basis and are therefore more likely to be able to give a representative view of their child’s behaviour across different contexts, compared to an observer who is only viewing the child on a single day [53]. Moreover, ADHD is related to novelty-seeking [98], and parent ratings may more accurately reflect their child’s behaviour in less novel, familiar environments, where some ADHD-related behaviours may be more likely to occur [99]. Despite the strengths of having a parent informant, the shared variance between parent ratings of their child’s behaviour at 10 months and 3 years should be taken into account when interpreting our findings, and may explain why the parent ratings were more strongly predictive of later ADHD-related behaviours than observer ratings in some of our models. Furthermore, neither the parent nor observer ratings were made blind to family history status. Future studies should include further objective, blinded measures of activity level alongside informant ratings of global behaviour. For example, Miller et al. (2020) [46] found that when infant behaviour was coded by observers who were blind to family history status, infants with a first degree relative with ADHD exhibited 153% more “out of seat behaviours”, compared to a control group. Objective methods such as actigraphy could also be informative in measuring infant activity level in prospective study designs [47]. However, in our study, we did not find increased head motion associated with having a family history of ADHD, suggesting that it may also be important to consider the context in which objective measures are implemented (both in terms of the setting, and experimental task). Indeed, in a sample of 2-year-olds, Ilott et al. (2010) [99] found that objective measures of activity level showed a greater genetic correlation with symptoms of ADHD when measured in the home than in the lab. Later in development, physiological markers of arousal may differ between individuals with and without ADHD during slower, less rewarding experimental tasks, but not in faster task conditions [100].

While we found that infant activity level associated with preschool ADHD traits at 3 years, we did not find the same relationship for infant attention. Infant attention measured both at the behavioural and neurocognitive level did not associate with preschool ADHD traits at 3 years. We preregistered our selection of neurocognitive measures of attention based on both what is known about the developing infant attention system, and neurocognitive differences that have been associated with the later ADHD phenotype (e.g., reaction time variability, elevated theta power). However, some research suggests that the pattern of neurocognitive markers associated with conditions such as ADHD or ASD may change over development [101]. Thus, we may need to look beyond the core phenotype of ADHD to identify the earliest alterations associated with later symptoms.

Furthermore, some studies have demonstrated low convergence between commonly used measures of infant attention [52,102,103]. In our sample, we also found generally low convergence between our selected measures of infant attention (Figure 2), especially across different modalities. Therefore, it is perhaps not surprising that we did not find longitudinal associations between measures of infant attention and later ADHD traits, given the inconsistent associations between these measures at the infant timepoint. One exception was peak look duration which showed a moderate association across both naturalistic toy play and an eye-tracking task, and also associated with frontal theta power, suggesting that this metric may be stable across different measurement techniques and contexts, and perhaps a promising measure to explore further (though see [52]). Of course, attention is multifaceted and the lack of significant correlations between measures does not necessarily mean that these measures are not assessing infant attention. Rather, they could be assessing different aspects of it. Nevertheless, these results highlight the need for further understanding of how different measures of infant attention relate to one another, as well as to later behaviour.

For triangulation, we further examined the association between infant activity level and emerging ADHD traits, by investigating associations with broader temperament dimensions related to the later ADHD phenotype at 3 years including impulsivity, attentional focusing, inhibitory control and activity level (measured using the CBQ). Though the CBQ is a validated and widely used measure of temperament in preschool aged children [87], our results should be interpreted in the context that we did not find high internal consistency for the subscales of interest in our sample. We found that infant activity level was predictive of inhibitory control and activity level at 3 years. It was also predictive of impulsivity (though this result did not survive correction for multiple testing). Infant activity did not predict attentional focusing at 3 years. This is consistent with findings that preschool activity level is predictive of later hyperactivity/impulsivity in adolescence, but not inattention [28]. Preschool ADHD is predominantly defined by hyperactivity/impulsivity [33] and some have suggested that the DSM-defined subtypes of ADHD are not applicable to preschoolers [104], with rates of the hyperactive-impulsive subtype of ADHD decreasing in older children, and the rates of the combined or inattentive subtype of ADHD increasing [33,105,106]. Furthermore, polygenic scores for ADHD may be associated with symptoms of hyperactivity and impulsivity (but not inattention) [107]. Perhaps the genetic likelihood design of this study was therefore better suited to investigate pathways to hyperactive/impulsive behaviours than inattention.

A limitation of our study is that this cohort has so far only been followed up to the age of 3 years. This allowed us to investigate the relationship between infant markers of attention and activity level, and preschool ADHD traits at age 3. However, while there is some evidence of continuity from the preschool years [18,19], ADHD is not typically diagnosed until middle childhood when the full ADHD phenotype is present, and high levels of hyperactivity or inattention are not uncommon in preschoolers. Furthermore, recent research indicates that certain infant neurocognitive markers of attention (peak look duration) may relate to ADHD traits in mid-childhood, but not in toddlerhood [58]. Longer term follow-up of our cohort of infants to middle childhood could help to discriminate between the subtypes of ADHD, and their relationship to measures of attention and activity level over the course of development.

An additional limitation of our study is that a subset of the infants included in this study received a 9-week attention training programme following their 10-month lab visit, as part of a randomised controlled trial [22]. Our primary analyses in the present study are unaffected by this; no infants completed any training sessions until after their 10-month lab visit. As follow-up analysis for the trial is ongoing, it is not yet possible to comment on whether the training may have affected developing attention skills between the 10-month and 3-year timepoints. This will be examined in the future. However, it is expected that, if anything, this would have lowered our power to detect longitudinal associations between the infant and early childhood measures in the present study.

## 5. Conclusions

Using a multi-method, pre-registered assessment protocol, we found no significant group differences in activity level or attention in 10-month-old infants with and without an elevated likelihood of ADHD. Limitations of the study were noted, including the possibility that we did not select the most sensitive measures of infant attention in this study. In secondary longitudinal analyses, parent and observer ratings of infant activity level at 10 months significantly predicted ADHD-related behaviours at 3 years, including preschool ADHD traits (CBCL), and broader temperament dimensions related to the ADHD phenotype (activity level, inhibitory control and impulsivity; CBQ). Our findings support previous findings that early differences in activity level may be apparent in the first year of life in children who later develop elevated ADHD traits. It is important to acknowledge that infant activity level varies in the population [47], and high levels of activity level are not uncommon in typically developing infants and preschoolers [108]. Thus, while further investigation of infant activity level could help us to understand some of the earliest developmental processes associated with ADHD, it is premature to suggest the use of infant activity level as an early screener for ADHD. It is likely that there are multiple developmental pathways that may lead to the ADHD phenotype [25,109], only some of which may involve elevated activity levels. Future work should focus on understanding how infant activity level may interact with other cognitive processes, and the environment, over the course of development to impact later outcomes.

## Figures and Tables

**Table 1:** Participant characteristics at the 10-month and 3-year laboratory visits.

		10-Month Visit		
	FH-ADHD	FH-ADHD and ASD	FH-ASD	FH-No ADHD/ASD
*N*	27	20	77	27
Age (days) M (SD)	324 (28)	320 (15)	320 (15)	322 (17)
Range	278–384	300–354	287–357	293–358
Sex	12 female	8 female	38 female	11 female
	15 male	12 male	39 male	16 male
Highest education level of primary caregiver ^a^	8 Secondary 10 Undergraduate 9 Postgraduate	11 Secondary 6 Undergraduate 2 Postgraduate	1 Primary 18 Secondary 38 Undergraduate 18 Postgraduate	2 Secondary 10 Undergraduate 14 Postgraduate
MSEL ELC ^b^				
M (SD)	85.04 (15.61)	84.90 (16.55)	88.03 (15.09)	88.89 (12.19)
Range	61–128	59–134	50–136	58–114
		**3-Year Visit**		
	FH-ADHD	FH-ADHD and ASD	FH-ASD	FH-No ADHD/ASD
N ^c^	21	14	57	21
Age (months)				
M (SD)	37.42 (2.93)	37.21 (1.63)	37.00 (1.17)	36.83 (1.82)
Range	36–49	36–41	36–42	35–43
Sex	9 female	4 female	30 female	9 female
	12 male	10 male	27 male	12 male
Highest education level of primary caregiver ^d^	5 Secondary 8 Undergraduate 8 Postgraduate	7 Secondary 4 Undergraduate 2 Postgraduate	1 Primary 14 Secondary 29 Undergraduate 11 Postgraduate	1 Secondary 7 Undergraduate 12 Postgraduate
MSEL ELC				
M (SD)	118.58 (20.37)	106.00 (20.65)	107.27 (19.24)	129.94 (11.41)
Range ^e^	66–144	65–131	75–141	109–146

^a^ Data missing for 4 participants (2 FH-ASD; 1 FH-No ADHD/ASD; 1 FH-ADHD and ASD). ^b^ Further information regarding MSEL administration is provided in SM3. Data is missing for one participant in the FH-ADHD group, and one participant in the FH-ASD group. ^c^ Demographics at 3 years are presented only for participants who had both valid data at the 10-month time point and data on the ADHD subscale of the CBCL at 3 years. Data at 3 years also includes three children with a half-sibling with ADHD. These infants were not included in the primary 10-month analyses due to not meeting eligibility criteria for the FH-ADHD group or the FH-No ADHD/ASD group. However, they were included in the longitudinal analyses. For these participants at 3 years, Age M (SD) = 37.33 (2.31); Sex = 3 male; Highest education of primary caregiver = 3 Secondary; MSEL ELC M (SD) = 99.67 (44.29). ^d^ Data missing for 4 participants (2 FH-ASD; 1 FH-No ADHD/ASD; 1 FH-ADHD and ASD). Data was collected at the 10-month timepoint. ^e^ Data missing for 11 participants (2 FH-ADHD; 6 FH-ASD; 3 FH-No ADHD/ASD). MSEL; Mullen Scales of Early Learning. The MSEL (Mullen, 1995) is a standardized measure that assesses developmental abilities across five subscales including: Fine Motor, Gross Motor, Visual Reception, Receptive Language and Expressive Language. FH, Family History; ADHD, Attention Deficit Hyperactivity Disorder; ASD, Autism Spectrum Disorder; ELC, Early Learning Composite.

**Table 2:** Effect of Family History of ADHD and/or ASD on infant activity level and attention.

Modality		Effect of FH-ADHD	Effect of FH-ASD	Interaction Effect of FH-ADHD and FH-ASD
Global ratings of attention (IBQ-R, observer ratings)	*F*	0.126	0.675	1.095
	*P*	0.882	0.511	0.338
	Partial η^2^	0.002	0.012	0.019
	Partial ω^2^	0.000	0.000	0.002
Global ratings of activity level (IBQ-R, observer ratings)	*F*	1.374	1.696	1.667
	*P*	0.257	0.188	0.193
	Partial η^2^	0.024	0.029	0.029
	Partial ω^2^	0.006	0.012	0.011
Behavioural measures of active attention (Total and peak)	*F*	0.052	2.917	0.230
	*P*	0.949	0.057	0.795
	Partial η^2^	0.001	0.040	0.003
	Partial ω^2^	0.000	0.026	0.000
Eye tracking measures of attention (Reaction time variability and peak look)	*F*	0.067	0.879	1.836
	*P*	0.936	0.418	0.164
	Partial η^2^	0.001	0.014	0.029
	Partial ω^2^	0.000	0.000	0.013
Physiological measures (Heart rate and head motion)	*F*	2.230	1.487	0.400
	*P*	0.114	0.232	0.672
	Partial η^2^	0.052	0.035	0.010
	Partial ω^2^	0.028	0.011	0.000
Neural measures (Mean frontal theta, theta change)	*F*	0.126	0.271	0.070
	*P*	0.882	0.763	0.932
	Partial η^2^	0.002	0.005	0.001
	Partial ω^2^	0.000	0.000	0.000

**Table 3:** Descriptive statistics for Child Behaviour Questionnaire (CBQ) and CBCL at 3 years for the full cohort ^a^.

	N	*M* (SD)	Range
CBCL ADHD subscale Total	116	4.30 (3.23)	0–12
CBQ Impulsivity	112	4.38 (0.92)	1.83–7.00
CBQ Inhibitory Control	113	4.42 (1.04)	1.00–6.50
CBQ Activity Level	114	4.85 (0.88)	2.71–6.71
CBQ Attentional Focusing	114	4.47 (0.95)	1.17–6.67

^a^ Data only shown for participants who also had valid data at the 10-month infant visit.

## Data Availability

Due to ethical and privacy restrictions, the data in this study is not publicly available. Data is available on request from the corresponding author subject to the data sharing policies of the BASIS/STAARS network (see www.basisnetwork.org).

## Supplementary Materials

The following are available online at https://www.mdpi.com/article/10.3390/brainsci11050524/s1.
