# Supplementary material for: Behavioural Measures of Infant Activity but Not Attention Associate with Later Preschool ADHD Traits
Source: Brain Sci. 2021 Apr 21;11(5):524. doi: 10.3390/brainsci11050524 (PMC8143002; doi:10.3390/brainsci11050524)
Supplement: Supplementary file 1 [file brainsci-11-00524-s001.zip › brainsci-1040737-SI.pdf]

Supplementary Materials

Table of Contents

SM1. Pre-registered analyses ('FH-ADHD' and 'FH-No ADHD/ASD' only)........................2
SM2. Data exclusions prior to analysis..3
SM3. MSEL Administration..3
SM4. Classification of family history status...4
SM5. Missing data...5
SM6. Active Attention Coding Scheme...6
SM7. Heart rate data processing..7
SM8. Head motion data processing...8
SM9. Frontal electrodes used for analyses..9
SM10. CBQ and CBCL at 3 years split by family history status.................................9
SM11. Infant activity level and temperament traits at 3 years....................................10
SM12. Results from univariate analyses..12
SM13. Effect of attention and activity on missing data..13
SM14. CBCL and sex at 3 years...14
Supplementary References...15

SM1. Pre-registered analyses ('FH-ADHD' and 'FH-No ADHD/ASD' only)

In the main manuscript we report the results of analyses conducted on data from infants with FH-ADHD, FH-ASD, FH-ADHD and ASD, and FH-No ADHD/ASD. This was an extension of our original preregistered plan to conduct comparisons between FH-ADHD and FH-No ADHD/ASD infants only (https://osf.io/kyc46). Below, we report the results of these pre-registered analyses. Consistent with the results presented in the main manuscript, a MANOVA revealed no significant effect of family history of ADHD on any of the behavioural or neurocognitive measures of activity level and attention at 10 months of age. This was the case both when all FH-ADHD infants were included, and when only FH-ADHD infants without a family history of ASD were included; see Table S1.

Table S1. Effect of FH-ADHD on infant activity level and attention

Modality		*All FH-ADHD infants (n=47)*	*FH-ADHD, excluding infants also with FH-ASD (n=27)*
Global ratings of attention (IBQ-R, observer ratings)	F	.382	.889
	p	.684	.418
	Partial η^2	.013	.040
	Partial ω^2	.000	.000
Global ratings of activity level (IBQ-R, observer ratings)	F	1.451	1.268
	p	.243	.292
	Partial η^2	.048	.058
	Partial ω^2	.015	.012
Behavioural measures of active attention (Total attention and peak attention during toy play)	F	0.054	.141
	p	.948	.869
	Partial η^2	.002	.006
	Partial ω^2	.000	.000
Eye tracking measures of attention (Reaction time variability and peak look)	F	.089	.550
	p	.915	.581
	Partial η^2	.003	.025
	Partial ω^2	.000	.000
Physiological measures (Heart rate and head motion)	F	1.096	2.087
	p	.344	.143
	Partial η^2	.053	.130
	Partial ω^2	.005	.065

Neural measures (Mean frontal theta, theta change)	*F*	0.146	.063
	p	.865	.939
	Partial η^2	.006	.003
	Partial ω^2	.000	.000

SM2. Data exclusions prior to analysis

Data for the present study comes from the first wave of participants in the STAARS study, which includes infants who attended their first visit to the laboratory for the project prior to June 2017. This includes a total of 164 infants. However, 13 of these infants were excluded from the analyses in the present study due to the reasons stated below:

Table S2. Reasons for exclusion prior to analysis

Reason for exclusion	*N*
Infant did not attend 10 month lab visit	10
Family history categorisation[a]	3

[a] This includes three infants with a half-sibling with an ADHD diagnosis. These infants were not included in the primary 10 month analyses due to not meeting eligibility criteria for the 'FH-ADHD' group or the 'FH-No ADHD/ASD' group. However, they *were* included in all secondary analyses, which included the full cohort.

SM3. MSEL Administration

The STAARS study is part of a larger European multi-site study [1]. As described in Begum Ali et al. (2020) [2], strict guidelines were used to administer and score the MSEL (to allow for replicability and consistency across sites). At the 10-month time point, only behaviours that were captured on camera and within the MSEL session were scored, so that they could be confirmed by a second/third researcher if necessary. For example, if an infant demonstrated babbling throughout the rest of the testing day (i.e., during another task or a lunch break), but not during the specific Mullen administration session, this was not scored as the infant being able to produce babbling sounds on the Expressive Language scale. To further ensure the fidelity of the scoring, a

second fully trained researcher watches the administration in real time (via a video feed) and consensus discussions take place after the testing session. These strict administration and scoring guidelines (although they are recommended in the Mullen manual) may not be those applied more broadly in the field, and thus may account for relatively poorer performance in this cohort at the 10 month timepoint relative to US norms. At the 3-year time point, scores are in the typical range (Table 1 in the main manuscript).

SM4. Classification of family history status

For siblings (6 years or older), a shortened adapted version of the Conners 3 [3] was used. Behaviours that parents reported as occurring either “often” or “frequently” were scored. All included children met a minimum threshold for inclusion of i) 6 ADHD symptoms on either the hyperactivity/impulsivity scale (consisting of item numbers: 3, 43, 45[54]*, 61, 69[99]*, 71, 93, 98, 104) or the inattention scale (consisting of item numbers: 2, 28, 35, 47, 68[79]*, 84, 95, 97, 101), and ii) a positive score on the impairment scale (at least 2 out of 3 impairment items, consisting of item numbers: 106, 107, 108).

For siblings (aged less than 6 years), a shortened adapted version of the Conners Early Childhood [4] form was used. Behaviours that parents reported as occurring either “often” or “frequently” were scored. All included children met a minimum threshold for inclusion of i) 9 ADHD symptoms on the inattention/hyperactivity scale (consisting of item numbers: B8, B12, B22, B34, B42, B47, B49, B55, B65, B72, B74), and ii) a positive score on the impairment scale (at least 2 out of 3 impairment items, consisting of item numbers: IM1, IM2, IM3).

For parents, a shortened adapted version of the Conners Adults ADHD Rating Scale (CAARS) [5], either self or observer report. Behaviours that parents reported as occurring

either “often” or “frequently” were scored. All included parents met a minimum threshold for inclusion of 5 ADHD symptoms on either the hyperactivity/impulsivity scale (consisting of item numbers: 2, 4, 6, 8, 16, 18, 22, 25, 27) or the inattention scale (consisting of item numbers: 1, 9, 13, 14, 19, 21, 26, 29, 30). Of note, the adult version of the Conners does not include impairment questions.

*Indicates that these two items were collapsed into a single question in the adapted screening form.

SM5. Missing data

For the 151 infants who were included in the primary analyses at the 10 month time point, missing data per measure is shown below. The following coding system is used:

**EX**	Data excluded, e.g. not enough valid trails
**NC**	Data not collected
**NR**	Questionnaire not returned
**NT**	Not enough time
**PR**	Parent refused
**TI**	Technical issue

Table S3.

**Measure**	**Reason Missing**	**N Missing**
IBQ-R Activity Level	NR	22
	EX	3
IBQ-R Duration of Orienting	NR	22
	EX	4
Observer Ratings (Attention)	NC	8
Observer Ratings (Activity)	NC	9
Lab-TAB Task Orientation Episode	NT	3
	TI	1
	EX	2
	NC	1
Gap overlap variability (Eye-tracking)	EX	3
	NC	4
Peak look face-pop out (Eye-tracking)	EX	5
	NC	18

Heart rate	EX	23
	NC	8
	TI	14
	NT	3
	PR	1
Head motion	NC	1
	EX	24
EEG Mean Frontal Theta	NC	9
	EX	6
	TI	1
EEG Theta Change	NC	9
	EX	32
	TI	1

SM6. Active Attention Coding Scheme

Table S4.

Manipulation of block

Proportion of time spent manipulating the blocks. Manipulation was defined as a frame in which the child was touching and/or holding the blocks. Manipulation does not include throwing the blocks off the table or mouthing the blocks.

Looking at blocks

Proportion of time spent looking at the blocks. Looking at the blocks was defined as the child visually fixating one or more of the blocks.

Active Attention

Active Attention was the proportion of time the child spent looking at and manipulating the blocks. This was identified through the overlap between the looking and manipulating codes (calculated within Mangold Interact).

SM7. Heart rate data processing

Heart rate data was collected using the RSPEC BioNomadix (BIOPAC Systems, Inc), attached to the MP150 amplifier. Three electrodes were placed in a lead-II position on the child's back and the transmitter was placed in the pocket of a one-piece baby suit designed for the study. Data was acquired in AcqKnowledge (version 4.4, BIOPAC Systems, Inc), sampled at either 100 or 1000 Hz with a 35 Hz low pass and 1 Hz high pass filter. A LED light, which sends a digital marker to AcqKnowledge, was used to sync the video and physiological data. The onset of the light and the LabTAB task were coded in Mangold and were used to find the start of the task in Acqknowledge. R-peaks were identified visually offline in AcqKnowledge, then exported into MATLAB. Up to three consecutive missing R-peaks were inserted by dividing the interval by the amount of missing peaks plus one. Average heart rate was taken over 30 second epochs. Any epochs with four or more consecutively missing peaks were marked as invalid. The total average heart rate for each child was the average heart rate of all valid epochs (maximum of six epochs during the three-minute LabTAB task).

SM8. Head motion data processing

Head motion was derived from the 3D coordinates of each eye, as reported by the eye tracker and down-sampled to 30Hz. Distance is the first derivative (the Euclidean distance between consecutive samples), and velocity is the second derivative. To normalise for quantity of valid, binocular samples (i.e. amount of clean data during which the infant attended to the screen) we used mean velocity as the derived variable to indicate amount of head motion. There are two major sources of error using this method. Firstly, whilst Tobii eye trackers acquire binocular data where possible, monocular samples are returned in situations where the eye tracker can only detect one eye. If the eye tracker misidentifies one eye (e.g. the left) as the other (e.g. the right), the mean eye position (the location in the centre of the eyes, approximately the bridge of the nose) will appear to "jump" by half the interocular distance. To avoid this, we discarded all monocular samples. Secondly, this process is subject to overestimation of distance due to measurement error (noise) between adjacent samples, and to correct this we first smoothed the raw data using robust local regression with a linear fit (RLOWESS). The precision of eye position estimates in our data differed according to the axis of measurement, with in general poorer precision in the z-axis (distance from head to eye tracker) than in the x- or y-axes. Additionally, precision differed between individuals. For these reasons we used different smoothing parameters (the span across which smoothing was applied) for each individual and for each axis. To find the most appropriate parameters without introducing bias we evaluated a range of spans from 233ms to 1000ms, then calculated the sum of squared errors (SSE) between the raw and the smoothed data, selecting a final span parameter to minimise SSE. To avoid over-fitting, we performed this process using k-fold cross validation with k=10. After smoothing, sample-by-sample velocity was derived and the mean velocity calculated for each trial.

SM9. Frontal electrodes used for analyses

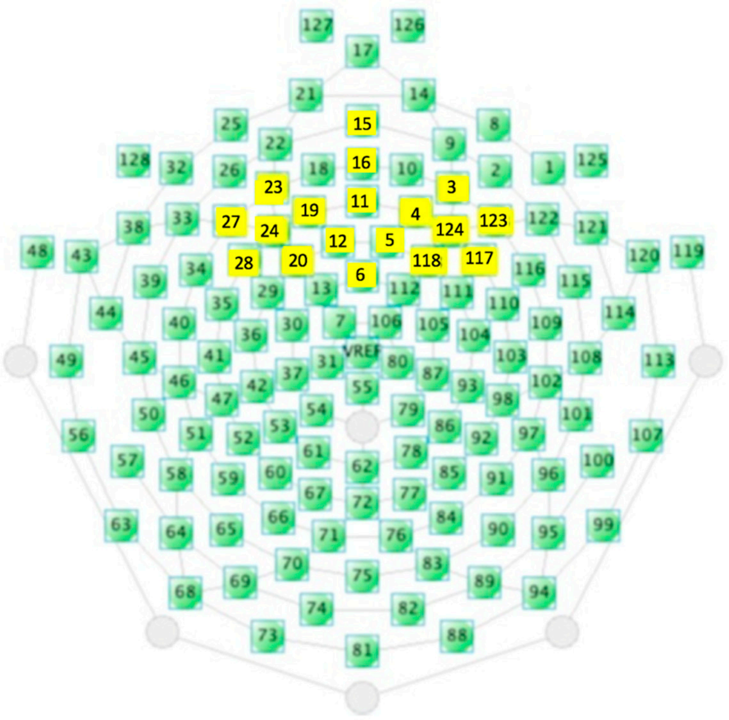

SM10. CBQ and CBCL at 3 years split by family history status

Table S5.

	**FH-ADHD**	**FH-ASD**	**FH-ADHD and ASD**	**FH No ADHD/ASD**
CBCL ADHD subscale Total M(SD)	4.48 (3.28)	4.30 (3.26)	5.79 (3.93)	3.05 (2.16)
CBQ Impulsivity M(SD)	4.67 (1.12)	4.25 (0.87)	4.73 (1.00)	4.31 (0.69)
CBQ Inhibitory Control M(SD)	4.47 (1.05)	4.43 (0.92)	3.85 (1.50)	4.84 (0.86)
CBQ Activity Level M(SD)	4.88 (1.06)	4.80 (0.84)	5.10 (0.97)	4.69 (0.76)
CBQ Attentional Focusing M(SD)	4.57 (0.99)	4.33 (0.84)	3.92 (1.17)	5.18 (0.72)

SM11. Infant activity level and temperament traits at 3 years

Scatterplots of infant activity level at 10 months and each CBQ outcome at 3 years are shown below for 1) impulsivity, 2) inhibitory control, 3) attentional focusing, and 4) activity level. Across all plots, the following key is used:

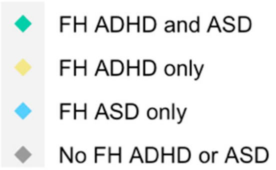

CBQ Impulsivity

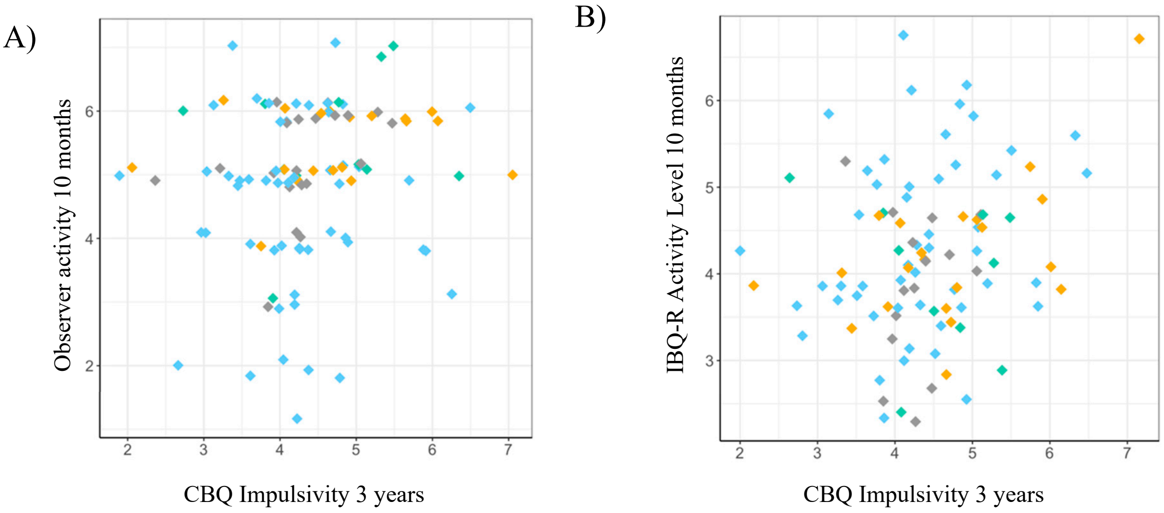

A) Observer ratings of infant activity level at 10 months on the y axis, and CBQ impulsivity at age 3 years on the x axis and, B) Parent ratings of infant activity level at 10 months (IBQ-R) on the y-axis, and CBQ impulsivity at age 3 years on the x axis, with jitter.

CBQ Inhibitory Control

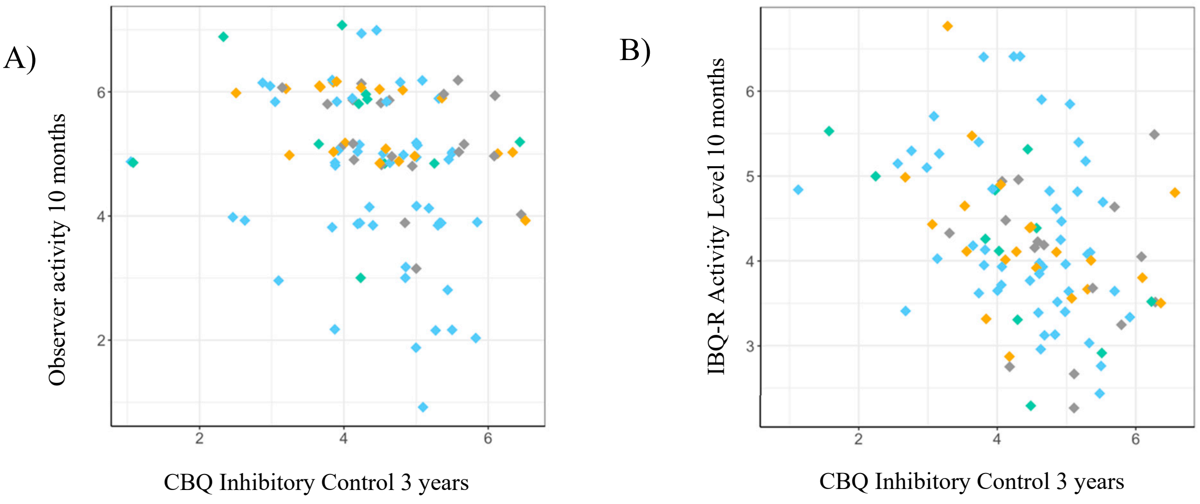

A) Observer ratings of infant activity level at 10 months on the y axis, and CBQ inhibitory control at age 3 years on the x axis and, B) Parent ratings of infant activity level at 10 months (IBQ-R) on the y-axis, and CBQ inhibitory control at age 3 years on the x axis, with jitter.

CBQ Attentional Focusing

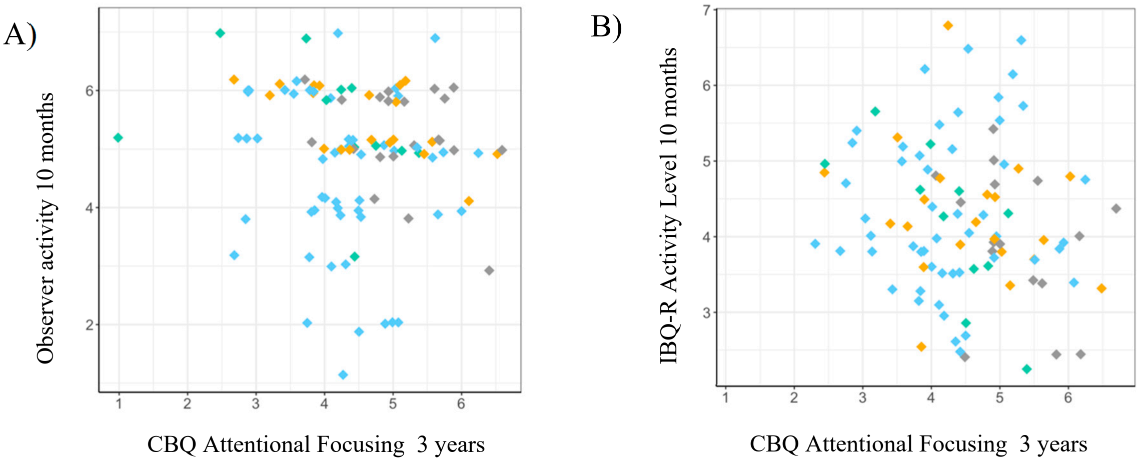

A) Observer ratings of infant activity level at 10 months on the y axis, and CBQ attentional focusing at age 3 years on the x axis and, B) Parent ratings of infant activity level at 10 months (IBQ-R) on the y-axis, and CBQ attentional focusing at age 3 years on the x axis, with jitter.

CBQ Activity Level

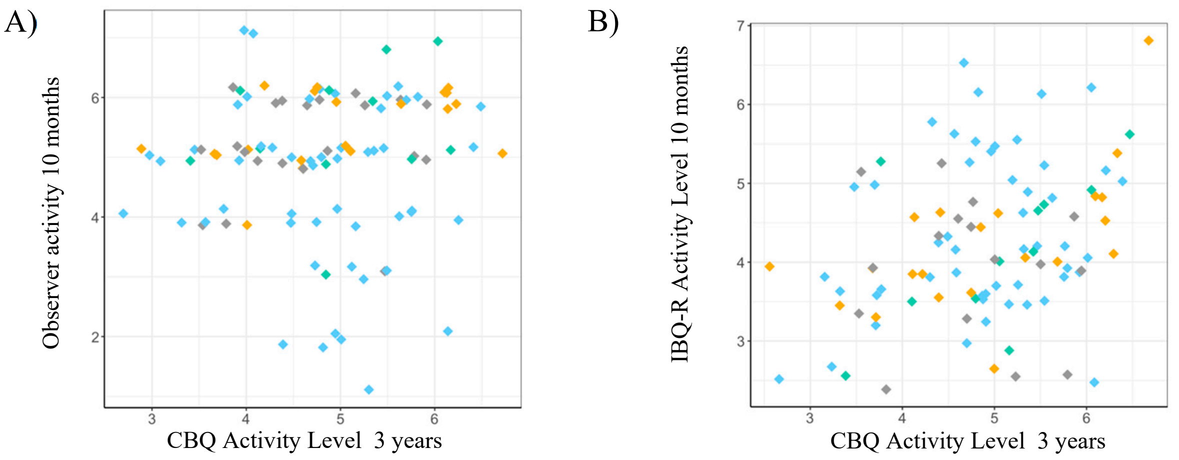

A) Observer ratings of infant activity level at 10 months on the y axis, and CBQ activity level at age 3 years on the x axis and, B) Parent ratings of infant activity level at 10 months (IBQ-R) on the y-axis, and CBQ activity level at age 3 years on the x axis, with jitter.

SM12. Results from univariate analyses

Table S6.

**Measures**		**Effect of FH-ADHD**	**Effect of FH-ASD**	**Interaction effect of FH-ADHD and FH-ASD**
IBQ-R Activity Level	*F*	1.016	2.137	1.099
	p	.316	.146	.297
	Partial η^2	.008	.017	.009
IBQ-R Duration of Orienting	*F*	.067	.968	.367
	p	.796	.327	.546
	Partial η^2	.001	.008	.003
Observer rated activity	*F*	1.509	2.708	1.356
	p	.221	.102	.246
	Partial η^2	.011	.019	.010
Observer rated attention	*F*	.357	2.419	1.525
	p	.551	.122	.219
	Partial η^2	.003	.017	.011
Peak active attention during play	*F*	.081	5.865	.296
	p	.776	.017*	.588
	Partial η^2	.001	.040	.002
Total active attention during play	*F*	.095	2.524	.006
	p	.759	.114	.940
	Partial η2	.001	.018	.000
	F	.052	.299	2.901

Peak look (Face Pop Out)	*p*	.820	.585	.091
	Partial η2	.000	.002	.023
Reaction time variability (Gap Overlap)	*F*	.047	.094	.007
	p	.829	.760	.932
	Partial η2	.000	.001	.000
Heart rate during play	*F*	1.289	1.631	.677
	p	.259	.205	.413
	Partial η2	.013	.016	.007
Head motion	*F*	3.413	.734	.430
	p	.067	.393	.513
	Partial η2	.027	.006	.004
Frontal theta change	*F*	.254	.273	.045
	p	.615	.603	.832
	Partial η2	.002	.003	.000
Mean frontal theta	*F*	.192	.805	.083
	p	.662	.371	.774
	Partial η2	.001	.006	.001

*In univariate analyses, there was a significant effect of FH-ASD on peak active attention during play. Infants with a family history of autism showed longer epochs of peak focused attention during play than infants without a family history of autism, F (1, 140) = 5.87, p = 0.017, η2 = .040. Of note, this univariate analysis included the same participants (and participant N) as the MANOVA that included this measure.

SM13. Effect of attention and activity on missing data

For attention, infants with scores in the upper quartile for the duration of orienting subscale of the IBQ-R were selected as the "high attention" group, and those with scores in the lower quartile were selected as the "low attention" group. For activity level, infants with scores in the upper quartile for the activity level subscale of the IBQ-R were selected as the "high activity" group, and those with scores in the lower quartile were selected as the "low activity" group. For infants who attended a 10-month visit at the laboratory, each infant was assigned a code of either 0 (data present and included in analysis), or 1 (data missing or excluded from analysis) for each experimental measure. Pearson Chi-Square tests indicated no significant effect of attention (high or low) or activity (high or low) on whether data was missing for any experimental measure, see Table S7 below.

Table S7.

Activity group and missing data	
**Experimental measure**	**Test statistics**
Active attention during play	χ (1) = 2.96, p = .09
Peak look (Face Pop Out)	χ (1) = 0.10, p = .92

Reaction Time Variability (Gap Overlap)	χ (1) = 0.16, p = .69
Heart rate during play	χ (1) = 0.91, p = .34
Head motion	χ (1) = 0.78, p = .38
Mean frontal theta	χ (1) = 0.10, p = .75
Theta change	χ (1) = 0.46, p = .50
Attention group and missing data	
**Experimental measure**	**Test statistics**
Active attention during play	χ (1) = 0.001, p = .98
Peak look (Face Pop Out)	χ (1) = 0.64, p = .42
Reaction Time Variability (Gap Overlap)	χ (1) = 0.32, p = .57
Heart rate during play	χ (1) = 0.03, p = .86
Head motion	χ (1) = 2.04, p = .15
Mean frontal theta	χ (1) = 0.10, p = .76
Theta change	χ (1) = 0.78, p = .38

SM14. CBCL and sex at 3 years

Total CBCL scores on the DSM ADHD subscale at 3 years did not differ significantly for males (M = 4.69) and females (M = 3.81), U = 1307.5, z = -1.61, p = .106, r = -0.15. The bar plot with scatter overlay below shows total CBCL scores on the DSM ADHD subscale at 3 years on the y-axis, for males (purple), and females (orange). Errors bars 95% CI.

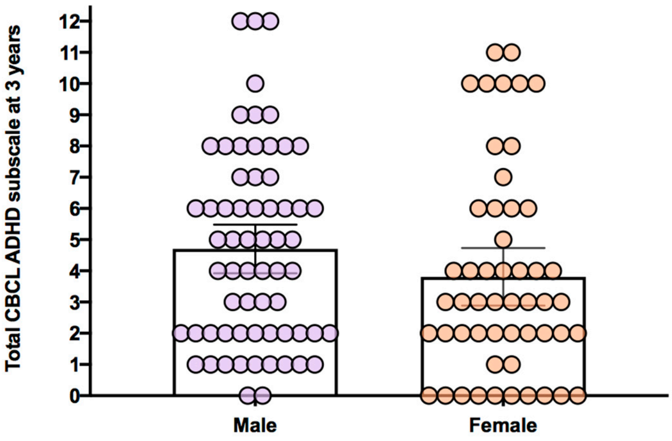

Supplementary References

1. Jones, E. J., Mason, L., Begum Ali, J., van den Boomen, C., Braukmann, R., Cauvet, E., ... & Bolte, S. (2019). Eurosibs: Towards robust measurement of infant neurocognitive predictors of autism across Europe. *Infant Behavior and Development*, *57*, 101316.

2. Begum Ali, J., Charman, T., Johnson, M. H., Jones, E. J., & BASIS/STAARS Team. (2020). Early motor differences in infants at elevated likelihood of Autism Spectrum Disorder and/or Attention Deficit Hyperactivity Disorder. *Journal of Autism and Developmental Disorders*, 1-18.

3. Conners CK. Conners 3rd edition. Toronto: Multi-Health Systems Inc; 2008.

4. Conners CK. Conners Early Childhood Manual. NY: Multi-Health Systems Inc; 2009.

5. Conners CK., Erdhardt, D., Sparrow, E. Conners Adults ADHD Ratings Scales (CAARS). NY: Multi-Health Systems Inc; 1999.
